# Long term follow up of growth in children with Congenital Adrenal Hyperplasia 21- Hydroxylase deficiency

**DOI:** 10.1186/1687-9856-2015-S1-P47

**Published:** 2015-04-28

**Authors:** Ruchi Parikh, Sudha Rao, Meena Desai, Ruchi Shah, Poonam Singh, Neha Dighe, Rajesh Joshi, Aparna Limaye

**Affiliations:** 1 Bai Jerbai Wadia Hospital for Children, Mumbai, Maharashtra, India

## 

Obesity and decreased final height are described in children with CAH 21OHD. Of 119 children (50 M, 69F;79 Salt Wasters(SW), 40 Simple Virilizers (SV)) diagnosed over 24 years, various growth parameters were studied in 43 children with regular follow up for 5 years or more.

Clinical data, anthropometry, genotype, hormonal and biochemical profile were evaluated at presentation. On follow up, growth and clinical characteristics, metabolic control (8am 17OH-Progesterone), bone age and replacement doses of gluco-corticoid (GC) and mineralo-corticoid (MC) were studied. Growth parameters were expressed as SDS. Obesity, defined as BMI SDS >/= 2 and short stature, defined as Ht SDS </= -2 were correlated with all the variables, using Unpaired T Test, Pearson Correlation and One way ANOVA Test.

43 children (16M, 27F; 32SW, 11SV; 36 mutations proven) had a mean duration of follow up of 11 +/- 4.14 years. At last follow up, Ht SDS was </= -2 in 27.9% cases (N=12/43: 4M, 8F; 9SW, 3SV). Age at onset of puberty (p=0.035), higher GC dose at presentation (>40mg/m2) (p=0.034) and at 3 years of age (p=0.047) and use of Hydrocortisone and/or Prednisolone (p=0.002) had a negative correlation with Ht SDS. 6 (13.95%) children (1M, 5F; 6SW) had achieved Adult Height (AH) SDS of -2.36 +/- 1.25, and AH SDS – TH SDS (Target Height) was -0.11 +/- 1.23. BMI SDS >/=2 found in 32.6% cases (N=14/43: 2M, 12F; 8SW, 6SV) correlated positively with 17 OHP values (p=0.013). Girls were heavier than boys (p=0.006). Adiposity rebound occurred at 4 years for both the genders. At the time of study analysis, Ht SDS showed a distinct shift to the left and BMI SDS, a distinct shift to the right of mean of the reference population as cited [1].

In the present series, there was a higher incidence of obesity (32.6%) but short stature was noted in 27.9% only. Aggressive lifestyle management, dietary control, optimizing dose of therapy (GC) and regular monitoring should be an integral part of long term management of patients with CAH 21OHD.
